# Information disorder and organic food purchasing behavior: A moderated mediation model

**DOI:** 10.3389/fnut.2022.939454

**Published:** 2022-07-19

**Authors:** Yan Zheng, Dayu Cao

**Affiliations:** ^1^School of Economics and Management, Jiangxi Agricultural University, Nanchang, China; ^2^School of Economics and Management, Jiangxi Rural Revitalization Strategy Research Institute, Jiangxi Agricultural University, Nanchang, China

**Keywords:** information disorder, involvement, moderated mediation model, attitude, organic food

## Abstract

On the one hand, fast social media and internet evolution has brought opportunities to the development of the organic food industry. On the other hand, the excessive utilization of social media and internet has also exerted some negative effects on consumers in terms of information disorder and hindered the industrial progression of organic foods. This study aimed to probe into the relationships between organic food information uncertainty, information search anxiety, information overload and purchase behavior under the mediating role of attitude and the moderating role of involvement, introducing the concept of information disorder in the context of this issue. The data (cross-sectional) of 620 organic food consumers in Jiangxi Province, China were subjected to SEM (structural equation modeling). The results showed that organic food information uncertainty and information search anxiety significantly affected attitude, and attitude had a positive impact on purchase behavior. In addition, attitude significantly mediated the effects of organic food information uncertainty and information search anxiety on purchase behavior. Moreover, the indirect relationship between organic food information uncertainty and purchase behavior was moderated by involvement.

## Introduction

Currently, an increasing number of enterprises are actively integrating ecological and environmental concerns into their economic activities while leading green consumption choices (1). In addition, increasing attention to health, safety, nutrition and environmental protection is reflected in consumers' consumption views (2–5). As a typical representative of green, healthy, safe and nutritious food (6–8), organic food has attracted increasing attention from consumers, causing the organic food industry to develop rapidly globally (9). In the case of China, organic food has an overall market value of around 10.2 billion euros in 2020, representing a 12-fold increase from 2009 levels, and China was already the world's fourth-largest organic food market in 2020 (10). Suggestively, the development potential of organic food market in China is promising. However, the development of this market is hindered by plenty of factors (6). For instance, information disorder (e.g., excessive organic food advertisements on the internet and social media, false organic food information dissemination, etc.) results in consumers being suspicious of the benefits of organic food (11), which weakens consumers' enthusiasm for purchasing organic food. As a result, China's consumption per head was measured as only about 7 euros, which is far below the global level of consumption per head (15.8 euros) (10). Consequently, the current study analyses factors impeding the organic food market development in China and seeks corresponding solutions, which is of significance in practical terms.

In the previous literature, the consumption of organic food has been explored extensively. Rana and Paul (9) pointed out that altruistic (e.g., environmental friendliness and ethical consumerism) and egoistic factors (e.g., health, quality and safety) are the main reasons for the organic food purchase of consumers. Apart from that, attitude was considered to be a vital variable in predicting organic food purchase behavior [e.g., (6, 12–14)]. Meanwhile, the information asymmetry in the organic food market has been effectively managed by the rapid progression of internet and evolution of diverse social media platforms, which stimulated the consumption of organic food (6). De-Magistris and Gracia (15) also confirmed that consumers' access to more organic food information through the internet or social media can encourage them to buy organic food. Nevertheless, the rapid social media and internet evolution has also brought some negative effects to consumers, especially in terms of information disorder (16).

Information disorder refers to the information barrier between a phenomenon and essence caused by inaccurate information (17). Wardle and Derakhshan (18) abstractly classified information disorder from the internet and social media into misinformation, disinformation and malinformation. Many scholars have referred to the negative effects of information disorder from the internet and social media as the media and internet's “dark side,” recognizing its tremendous hazard to the consumer health and happiness (19, 20). Moreover, such “dark side” has been linked to issues such as false news dissemination (21), information search anxiety (22, 23), perceived information overload (24, 25), and information uncertainty (26).

Since the internet and social media have become the technologies representing today's era (27), addressing consumers' information disorder regarding organic food may be of great significance in promoting organic food consumption. Nonetheless, there have been scarce studies emphasizing the association of information disorder with organic food consumption. Kushwah et al. (28) constructed an innovation resistance framework and tried to use consumers' organic food involvement to moderate the relationship between innovation barriers (i.e., value, risk and image barriers) and organic food purchase behavior. However, the authors only considered the purchase barriers brought by organic food as an innovative product for consumers and did not consider information disorder barriers affecting consumers through technologies such as the internet and social media. In order to deal with the existing gap, the current study probes into the relationships between information disorder (i.e., organic food information uncertainty, information search anxiety, information overload) and purchase behavior by setting attitude as the mediating variable and involvement as the moderating variable, introducing the concept of information disorder.

In the present study, three major contributions are attempted. Firstly, the relationship of information disorder and organic food purchase behavior has seldom been assessed in the prior literature. To address this gap in research, this paper introduces the concept of information disorder and divides it into three dimensions: organic food information uncertainty, information search anxiety and information overload. The paper explores the relationships between organic food information uncertainty, information search anxiety, information overload and purchasing behavior, offering a possible enrichment to the extant literature concerning organic food purchase. Regarding the second contribution, this paper draws upon information disorder perspectives and evaluates the indirect impact of information disorder on organic purchase behavior by formulating a novel attitude-dependent theoretical framework. In doing so, this study offers a more holistic view about how information disorder influences organic purchase behavior. Finally, the current study focuses on the moderating effect of involvement. Furthermore, this study explores whether involvement moderates the indirect effect of information disorder on purchase behavior through attitude by constructing a moderated mediation model. In accordance with the obtained results, the indirect relationship between organic food information uncertainty and purchase behavior is moderated by involvement, which may serve as valuable guidance for organic sellers, organic producers and even public departments.

## Theoretical framework and hypotheses

### Organic food information uncertainty, information search anxiety, information overload and attitude

Floridi proposed the concept of “information disorder” in 1996 and pointed out that information disorder is an information barrier between phenomenon and essence caused by inaccurate information (17). Information disorder from the internet and social media was abstractly classified into misinformation, disinformation and malinformation (18). Misinformation indicates the information that is incorrect but not intentionally created to cause harm; disinformation refers to information that is incorrect and deliberately created to harm individuals, social groups, organizations or countries; and malinformation may be based on real information, but it is used to cause harm to individuals, organizations or countries, especially to publish information that should be kept private in the public domain. Many studies pointed out that information disorder from the internet and social media poses a great threat to the wellbeing of consumers (19, 20).

Recently, information disorder has been extensively studied in the fields of e-commerce, library and information science and enterprise management (20, 24, 27, 29, 30). For example, after the outbreak of COVID-19, information disorder from social media and the internet aroused excessive reaction and unusual buying behaviors of consumers, including toilet paper hoarding and food storage (24, 30). Social media and the internet have become the technologies representing today's era (27). Therefore, improving the information disorder of consumers regarding organic food may be of great significance in promoting the industrial progression of organic foods. Hence, in this study, the concept of information disorder is incorporated into the realm of consumer behavior.

Information disorder has been associated with problems such as information search anxiety (22, 23), perceived information overload (24, 25), and information uncertainty (26). Therefore, this paper divides information disorder into three dimensions: organic food information uncertainty, information search anxiety, and information overload. Janssen and Hamm (31) argued that due to uncertainty about organic food-related information, distinguishing the credence attributes and criteria of organic food from those of traditional food becomes more challenging for consumers. Moreover, Teng and Lu (8) confirmed that uncertainty impacts the consumer attitudes toward organic food adversely. In addition, Mai et al. (32) believed that information overload is negatively associated with consumers' attitudes. Erfanmanesh et al. (22) showed that information search anxiety has always been regarded as a psychological disorder that has an important influence on people's cognition, attitudes and behavior. Consequently, the hypotheses are the following:

H1a. Organic food information uncertainty impacts the purchase behavior adversely.
H1b. Information search anxiety exerts a negative effect on purchase behavior.
H1c. Information overload exerts a negative effect on purchase behavior.

### Attitude and purchase behavior

Attitude refers to be an association in memory between an object and an evaluation; the association may vary in strength, and therefore, in accessibility (33). The more accessible attitude is in memory, the more likely it is that the individual considers the attitude object evaluatively when he/she encounters it (33). Attitude is considered to be a critical variable to predict organic purchase behavior (6, 13). Bravo et al. (34) pointed out that consumers are more prone to purchase organic food when their attitude about such food is positive. Moreover, Dean et al. (35) proved that when the consumers' attitude toward organic food is positive, this attitude is reflected in their buying behavior. Nevertheless, some studies have found inconsistency between consumers' attitude and buying behavior concerning organic food; that is, when the organic food attitude of consumers is positive, this attitude does not affect their purchase behavior (36, 37). The present study assumes that attitude is a predictor of organic food purchase behavior. Thus, it is proposed that:

H2. Attitude impacts the purchase behavior in a positive way.

### The mediating effect of attitude

Pagiaslis and Krontalis (38) studied the association of motives for organic food consumption with the buying behavior using attitudes as a mediator. The results showed that consumers' attitudes mediate between the consumption motives and the buying behavior for organic foods. Besides, Pham et al. (39) found that attitude mediates the media information's impact on the purchase behavior of organic foods, and Liu et al. (6) found that attitude mediates the association of faint signals with organic purchasing behavior. Therefore, it is hypothesized that attitude connects the association of buying behavior with the organic food information uncertainty, information search anxiety and information overload as a mediator. Accordingly, the following hypotheses is tested:

H3a. Attitude mediates the effect of organic food information uncertainty on purchase behavior.
H3b. Attitude mediates the effect of information search anxiety on purchase behavior.
H3c. Attitude mediates the effect of information overload on purchase behavior.

### The moderating impact of involvement

Consumer involvement refers to the time and energy consumers spend searching for and processing information related to a product (40). Consumers' involvement with a product will eventually affect consumers' decision-making regarding the product (40). Involvement with organic food was considered to be an important factor affecting consumers' purchase behavior (41). Previous studies have indicated that the attitudes toward organic foods or experiences in organic food purchase varied greatly among individuals with different levels of involvement with organic food (42, 43). Squires et al. (43) pointed out that organic food consumers with high involvement exert a more positive attitude toward the environment and health than organic food consumers with low involvement. Moreover, Kushwah et al. (28) argued that consumers' organic food involvement moderates the effects of value barriers on organic food purchase behavior. Thus, presumably, involvement is a moderator connecting the information disorder–attitude association, which in turn affects purchase behavior. Accordingly, it is postulated that:

H4a-c. Involvement moderates the indirect effect of organic food information uncertainty as well as information search anxiety and information overload on purchase behavior through attitude.

**Figure 1:**
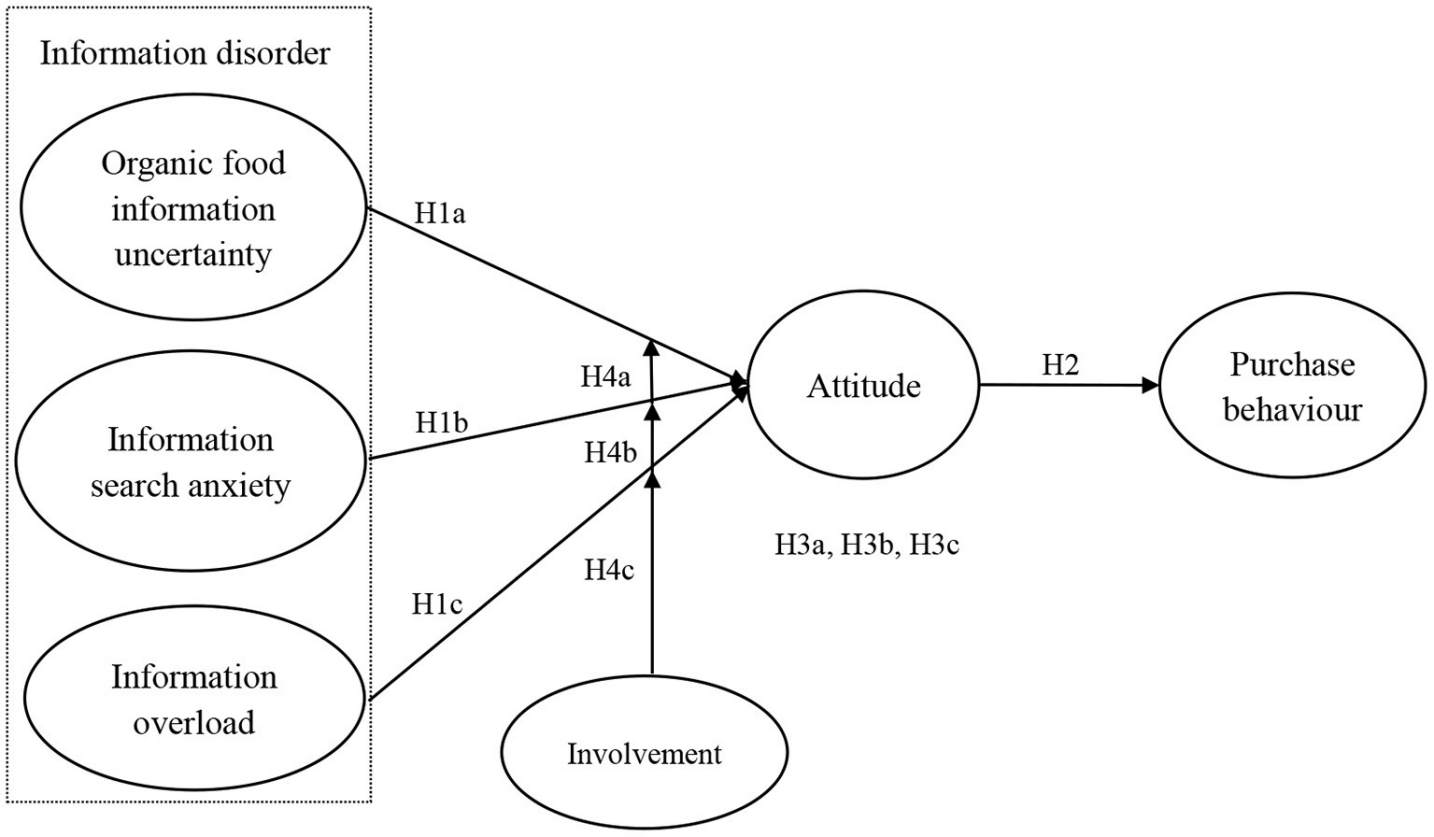
The hypothesized model.

Figure 1 illustrates the model hypothesized on the basis of foregoing discussion.

## Methodology

### Sample

**Figure 2:**
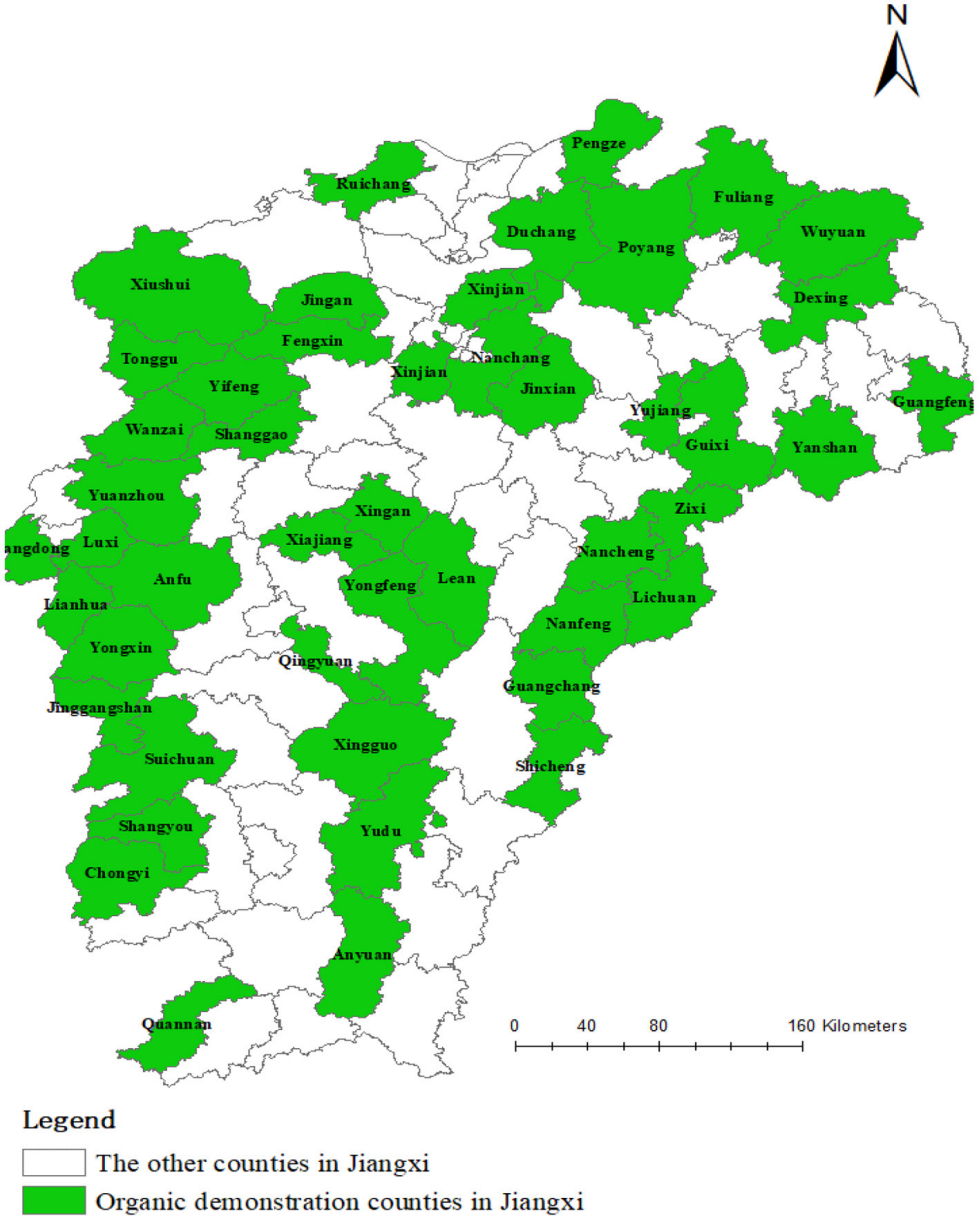
Demonstration counties of organic food in Jiangxi Province.

Jiangxi Province is one of the first provinces in China to be included in the construction of the first demonstration area of ecological civilization. To comprehensively improve the development of green agricultural products, Jiangxi Province selected five groups of 46 organic agricultural product demonstration counties from 2015 to 2019 (see Figure 2). In addition, in 2016, the Ministry of Agriculture of China listed Jiangxi Province as the only “pilot province of organic agricultural product demonstration base” in China. Therefore, this study used Jiangxi Province as the research area.

After defining the research area, stratified sampling was used to select the sample counties. First, according to the urban residents' disposable income per head in 2019, the above 46 counties were divided into three levels (see Appendix A). Namely, urban residents with disposable income per head less than 30,000 yuan were allocated to the low-income stratum; those with per capita disposable incomes of between 30,000 and 35,000 yuan were classified under the middle-income stratum; and those with per capita disposable incomes of above 35,000 yuan were classified under the high-income stratum. Then, two counties from each income stratum were selected as sample counties. Moreover, at least one sample county in each group of demonstration counties was ensured. Therefore, Nanchang, Xinjian, Fuliang, Jingan, Zixi and Poyang were ultimately selected as sample counties.

### Data collection

First, 30 questionnaires were distributed in Nanchang in September 2020 to conduct a preliminary survey, so that the questionnaire could be comprehensible. After the presurvey, the questionnaire was modified appropriately. Then, from October 2020 to December 2020, a formal questionnaire survey was conducted in the sample counties, including Nanchang, Xinjian, Fuliang, Jingan, Zixi and Poyang. The survey sites were located near large local supermarkets, and the questionnaire was randomly distributed. A total of 125 questionnaires per county were distributed, summing up 750 questionnaires. After eliminating invalid questionnaires (i.e., incomplete questionnaires, questionnaires with uniform answers, and questionnaires with obvious logical errors among answers), 620 usable responses were ultimately obtained.

As shown in Table 1, the sample included 50.5% male and 49.5% female respondents. Most of respondents (78.4%) were aged between 18 and 40. A total of 348 respondents (56.1%) had a junior high school education or below. There were 427 respondents (68.9%) with a monthly income per head exceeding RMB 5,000 yuan in the sample.

**Table 1:** Demographics of respondents (*N* = 620).

		***N***	**%**
1. Gender	Male	313	50.5
	Female	307	49.5
2. Age	18–30	261	42.1
	31–40	225	36.3
	41–50	116	18.7
	>50	18	2.9
3. Education	Below junior high school	110	17.7
	Junior high school	238	38.4
	High school or technical secondary school	146	23.5
	Junior college or undergraduate	118	19.0
	Postgraduate and above	8	1.3
4. Income (RMB/month)	≤ 3,000	67	10.8
	3,001–5,000	126	20.3
	5,001–8,000	215	34.7
	8,001–12,000	143	23.1
	>¥12,000	69	11.1

*¥ indicates RMB*.

### Measures

All constructs in the hypothesized model were measured with multiple-item scales that were validated in previous studies. A few minor modifications were made to the measures to ensure that they had face validity in the current research context. All the measures were evaluated using a five-point Likert-type scale ranging from 1 to 5, which indicated the degree from low to high (Table 2). For the purpose of determining organic food information uncertainty, 3 items from Teng and Lu's (8) study were used. For determination of information search anxiety, 3 items from Erfanmanesh et al. (22) scale were utilized. Information overload was determined with a 3-item scale taken from Bermes (24). For attitude determination, 4 items from Dean et al. (35, 44) were employed. Three items for involvement measurement from Teng and Lu (8) were adapted. Purchase behavior was measured with four items taken from Singh and Verma (45, 46).

**Table 2:** Confirmatory factor analysis properties.

**Construct and items**	**Std. factor**	**CR**	**AVE**
	**loadings**		
**Organic food information uncertainty (Cronbach's alpha** **=** **0.923)**			
1. I'm not sure of information about organic food.	0.896	0.923	0.801
2. Label information of organic food leads me to be unsure of the best choice for me.	0.892		
3. I have no confidence in evaluating organic and conventional food.	0.896		
**Information search anxiety (Cronbach's alpha** **=** **0.907)**			
1. I feel anxious when resources found while search for information on organic food are irrelevant.	0.916	0.909	0.769
2. Judging the quality of organic food information found through mobile phones or computers makes me anxious.	0.892		
3. I am worried about not being able to find the information I need when searching for information on organic food.	0.819		
**Information overload (Cronbach's alpha** **=** **0.890)**			
1. There is more information on organic food than I can digest.	0.895	0.891	0.732
2. The information on organic food overwhelms me.	0.820		
3. It is difficult for me to focus on essential information on organic food.	0.851		
**Attitude (Cronbach's alpha** **=** **0.815)**			
1. I think organic food is more nutritious than non-organic food.	0.675	0.806	0.511
2. I think organic food is safer than non-organic food.	0.781		
3. I think organic food is healthier than non-organic food.	0.694		
4.I think organic food is of higher quality than non-organic food.	0.704		
**Involvement (Cronbach's alpha** **=** **0.859)**			
1. I'm very familiar with organic food.	0.837	0.859	0.671
2. I'm highly involved in searching for and reading information about organic food.	0.809		
3. I have always been interested in organic food.	0.811		
**Purchase behavior (Cronbach's alpha** **=** **0.849)**			
1. How often do you buy organic food from an organic food store or supermarket?	0.790	0.85	0.587
2. How often do you buy organic food from an organic food demonstration base?	0.732		
3. How often do you buy organic food online?	0.785		
4. How often do you buy organic food from a community-group buying platform?	0.757		

### Control variables

This study controls for gender, education, age and income because former researches have proven that these variables affect organic food purchase behavior (47, 48).

### Research method

The hypothesized model was tested using structural equation modeling (SEM). By applying CB-SEM (covariance-based structural equation modeling), the data analysis followed many recent studies on organic food consumption (3, 6, 28, 36), as the study's objective was to test the proposed hypotheses rather than engage in theoretical construction. Moreover, SEM can depict concrete interrelationships between exogenous and endogenous variables, thus determining the exact effect strengths while controlling for measurement errors (49).

Data analysis was carried out using AMOS 24.0. It was confirmed that the measurement items were normally distributed. Then, CB-SEM through the 2-stage strategy SEM approach recommended by Anderson and Gerbing (50) was applied with confirmatory factor analysis (CFA) for the measurement model performed first, followed by the assessment of the ability of the structural model to answer the hypotheses. In addition, the moderated mediated effects of involvement on purchase behavior through attitude were tested using Hayes's PROCESS macro with SPSS 23.0.

## Results

### Common method variance

Harman's one-factor test was employed for examining whether the common-method variance harms the research as per prior procedure (51). As demonstrated by the results, no appearance of dominant factor was noted, and the covariance proportion explained by the first factor was considerably beneath 50%, the recommended threshold. This implies that the common-method variance is acceptable.

### Reliability and validity analysis

A good fitting model (χ^2^ = 429.009; df = 112; χ^2^/df = 3.83; GFI = 0.926; CFI =0.955; AGFI =0.899; RMSEA = 0.068) was returned by the measurement model (52, 53). The model measured internal reliability, as well as the convergent and discriminant validities. For this study constructs, both the CR (composite reliability) and Cronbach's alpha values were over 0.7, denoting an acceptable level of internal reliability (54) (see Table 2). In addition, the values of the Average variance extracted (AVE) were over 0.5, the recommended threshold (55), while the items' standardized factor loadings exceeded 0.6, the threshold value (52), providing support for convergent validity. Moreover, the inter-construct correlation coefficients were all beneath the square root of the AVE, which offers support for the discriminant validity (55) (see Table 3).

**Table 3:** Means, standard deviations and correlations.

	**Mean**	**SD**	**1**	**2**	**3**	**4**	**5**	**6**
1. Organic food information uncertainty	3.477	1.143	**0.895**					
2. Information search anxiety	3.542	1.107	0.590**	**0.877**				
3. Information overload	3.459	1.085	0.698**	0.691**	**0.856**			
4. Involvement	4.005	0.871	−0.239**	−0.304**	−0.258**	**0.819**		
5. Attitude	3.722	0.895	−0.328**	−0.326**	−0.263**	0.620**	**0.715**	
6. Purchase behavior	3.736	0.796	−0.582**	−0.459**	−0.510**	0.446**	0.546**	**0.766**

^**^p < 0.01; N = 620. Diagonal values in bold are the square root of the AVEs.

### Hypothesis testing

This study adopted structural equation modeling to examine the hypothesized relationships. The resulting fit indices of the structural model were measured as χ^2^ = 671.3; df = 182; χ^2^/df = 3.688; GFI = 0.908; CFI =0.932; AGFI = 0.883; RMSEA = 0.066, which indicated a good fitting model. Meanwhile, the model explained 51.2% of the variance of purchase behavior.

**Figure 3:**
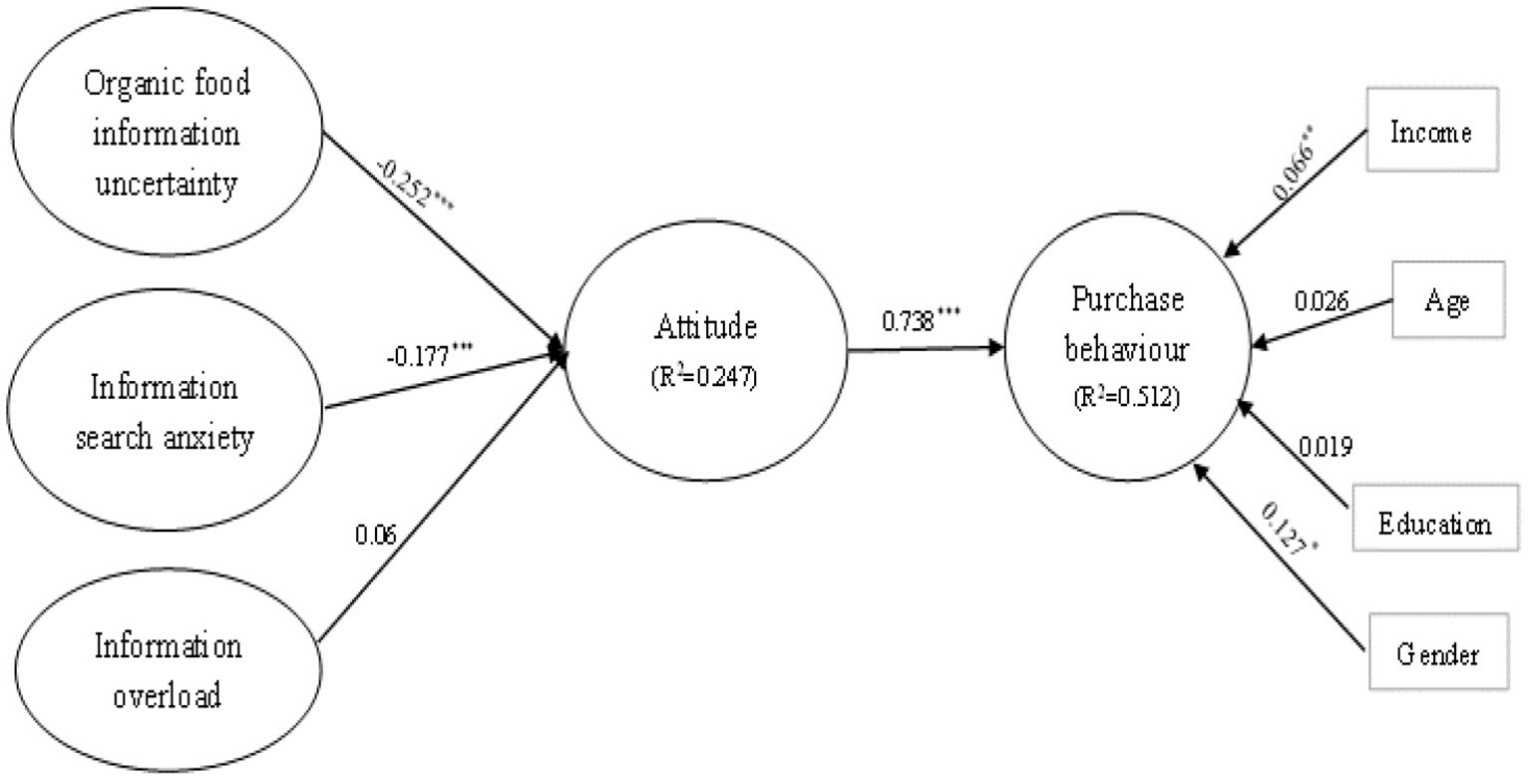
Tested model.

In accordance with Table 4 and Figure 3, the findings of hypothesis testing suggest the support of three of the hypotheses (H1a, H1b and H2). Notably, organic food information uncertainty (H1a: β= −0.252, *p* < 0.001) and information search anxiety (H1b: β = −0.177, *p* < 0.001) had a significantly negative effect on attitude. Therefore, H1a and H1b were supported. In addition, attitude was reported to impact the purchase behavior in a prominently positive way (H2: β = 0.738, *p* < 0.001), supporting H2. Nonetheless, information overload was discovered to have no obvious influences on attitude.

**Table 4:** Hypothesis test results.

**Hypothesis**	**Path**	**β**	**S.E**.	***T*-value**	***P*-value**	**Supported**
H1a	Organic food information uncertainty → Attitude	−0.252	0.052	−4.884	***	Yes
H1b	Information search anxiety → Attitude	−0.177	0.048	−3.672	***	Yes
H1c	Information overload → Attitude	0.06	0.062	0.962	0.336	No
H2	Attitude → Purchase behavior	0.738	0.057	12.865	***	Yes

^***^p < 0.001; N = 620.

In order to test the mediating effects of the independent variable through the mediator, bias-corrected percentile bootstrapping and percentile bootstrapping were performed at a 95% confidence interval with 5,000 bootstrap samples (56, 57). As presented in Table 5, the indirect effect of organic food information uncertainty on purchase behavior was significant (Bias-corrected percentile 95% confidence interval = −0.278, −0.101; Percentile 95% confidence interval = −0.274, −0.099), indicating that attitude mediated the relationship between organic food information uncertainty and purchase behavior, supporting H3a. Furthermore, information search anxiety had an indirect and significant impact on purchase behavior (Bias-corrected percentile 95% confidence interval = −0.210, −0.053; Percentile 95% confidence interval = −0.211, −0.054), indicating that attitude acted as a mediator between the anxiety of information search and the buying behavior, supporting H3b. However, attitude did not mediate the relationship between information overload and purchase behavior.

**Table 5:** Mediation test results.

	**Point**	**Product of**		**Bootstrapping**					
	**Estimate**	**coefficients**		**Bias-corrected percentile 95% CI**			**Percentile 95% CI**		
		**S.E**.	***Z***	**Lower**	**Upper**	**Two-tailed** **significance**	**Lower**	**Upper**	**Two-tailed** **significance**
OFIU → ATT → PB	−0.186	0.045	−4.133	−0.278	−0.101	***	−0.274	−0.099	***
ISA → ATT → PB	−0.131	0.040	−3.275	−0.210	−0.053	**	−0.211	−0.054	**
IO → ATT → PB	0.044	0.051	0.863	−0.056	0.147	0.388	−0.054	0.147	0.381

Estimation of 5,000 bootstrap sample; ^***^p < 0.001; ^**^p < 0.01. OFIU, organic food information uncertainty; ISA, information search anxiety; IO, information overload; ATT, attitude; PB, purchase behavior.

The hypothesized model proposed that involvement moderated the effects of organic food information uncertainty, information search anxiety and information overload on attitude, which in turn affected purchase behavior. The outcomes based on the first-stage model (58) showed that involvement moderated the effects of organic food information uncertainty on attitude (Table 6). Moreover, the conditional indirect impact of organic food information uncertainty on purchase behavior through attitude was negative and significant at both low levels (95% confidence interval = −0.318, −0.147) and high levels (95% confidence interval = −0.179, −0.015) of involvement. Hence, as powerfully evidenced by these outcomes, the mediator role of attitude was moderated by involvement.

**Table 6:** Conditional indirect effects on purchase behavior.

**Factor and statistic**	**Attitude**			**Purchase behavior**
	**OFIU**	**ISA**	**IO**	
**(A) Regression coefficients, first-stage moderation model**				
Gender	−0.061	−0.052	−0.054	0.075
Age	−0.042	−0.053	−0.054	−0.026
Education	−0.017	−0.016	−0.014	0.009
Income	−0.008	0.001	0.001	0.038
OFIU	−0.165***	−0.160***	−0.161***	−0.226***
ISA	−0.081*	−0.063	−0.073*	−0.013
IO	0.069	0.069	0.073	−0.120***
Involvement	0.574***	0.606***	0.596***	
OFIU * Involvement	0.077**			
ISA * Involvement		−0.049		
IO * Involvement			−0.036	
Attitude				0.348***
*R* ^2^	0.434	0.430	0.429	0.502
		**OFIU**		
**Moderator**	**Level**	**Conditional indirect effect**	**S.E**.	**Percentile 95% CI**
**(B) Conditional indirect effect of involvement on purchase behavior through attitude**				
Involvement	Low	−0.232	0.044	(−0.318, −0.147)
	High	−0.097	0.042	(−0.179, −0.015)

Estimation of 5,000 bootstrap samples; *p < 0.05; **p < 0.01; ***p < 0.001. OFIU, organic food information uncertainty; ISA, information search anxiety; IO, information overload.

**Figure 4:**
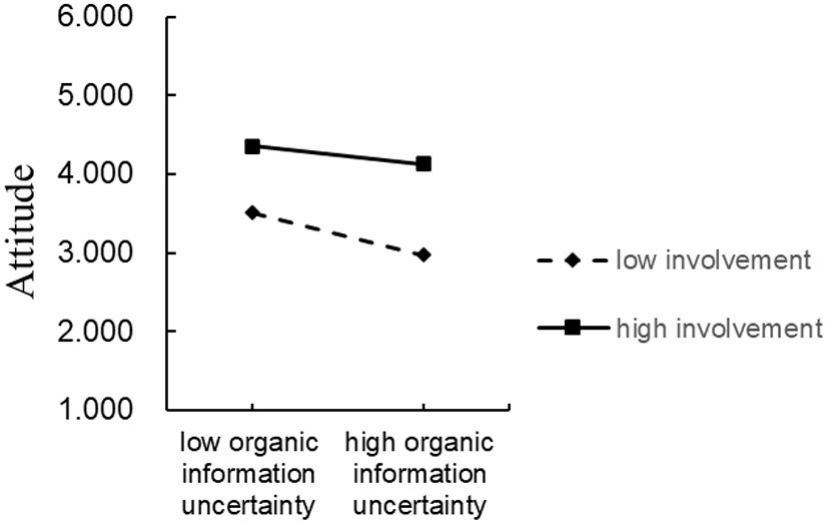
Interaction between organic food information uncertainty and involvement.

To further interpret the moderating effect of involvement, the interactive effects were depicted (Figure 4), which illustrated that involvement weakened the negative effect of organic food information uncertainty on attitude. The slope between organic food information uncertainty and attitude was significantly negative when the consumers were lowly involved (β = −0.232 *p* < 0.001) and negatively significant when the consumers were highly involved (β = −0.097, *p* < 0.05).

## Discussion and conclusion

In the field of organic consumption, there has been little research to examine the relationship between information disorder and organic food consumption. The current research bridges this study gap. In this study, the relationships between information disorder (i.e., organic food information uncertainty, information search anxiety, and information overload) and purchase behavior were explored by setting attitude as the mediator and involvement as the moderator, introducing the concept of information disorder. A hypothesized model was developed and adopted structural equation modeling and Hayes's PROCESS macro analysis to verify the hypotheses.

### Discussion of key findings

Firstly, in terms of information disorder, the findings indicated that organic food information uncertainty and information search anxiety had significant and negative influences on attitude. Consequently, the research findings offer evidence for the negative influences of information disorder in the field of organic food consumption. While previous empirical evidence on the impacts of information disorder in the organic food consumption realm is scarce, the present result agrees with prior studies in other domains (e.g., library and information science), which claimed that individuals with high information search anxiety have a negative attitude toward searched information (22). Nevertheless, information overload had no significant effects on attitude. This may be because organic food is a non-mainstream product in China, and consumers do not pay enough attention to organic food. In addition, the organic food consumption per head in China was measured as only about 7 euros, representing beneath half the global level of consumption per head (15.8 euros) (10). This may explain why information overload had no significant influences on attitude.

Secondly, attitude significantly mediated the effects of information disorder (organic food information uncertainty and information search anxiety) on the purchasing behavior. These findings are consistent with prior research, which suggested that attitude mediates the effect of media information on organic food purchase behavior (39). In addition, the study results revealed that attitude has a significantly positive effect on purchase behavior. That is, the enhanced attitude of consumers toward organic food can promote the purchase of organic food, which is in consistence with previous literature (6, 13). Nevertheless, attitude did not mediate the relationship between information overload and purchase behavior. This may be because the mediatory effect holds only when the mediator variable was influenced directly by the independent variable (59). But the study results found that the independent variable (information overload) had no direct influence on the mediating variable (attitude). Therefore, this may explain why attitude did not mediate the relationship between information overload and purchase behavior.

Finally, the results indicated that involvement moderated the effects of organic food information uncertainty on attitude. That is, involvement weakened the negative effect of organic food information uncertainty on attitude. These findings are consistent with prior research, which suggested that involvement weakened the negative effect of value barriers on organic purchase behavior (28). In addition, the study findings indicated that involvement moderates the indirect effect of organic food information uncertainty on purchase behavior through attitude. However, involvement had no moderating effect on other information disorders. It could be that the relationship between organic buyers and organic sellers is mainly based on trust (3). Meanwhile, organic food information uncertainty is the main factor preventing consumers from purchasing organic food (31). As such, consumers with high involvement might be better motivated to purchase organic foods because they trust organic food.

### Theoretical implications

In this study, the relationships between information disorder (i.e., organic food information uncertainty, information search anxiety, information overload) and purchase behavior were assessed by setting attitude as the mediator and involvement as the moderator. In general, the current study enriches the existing literature on organic food consumption in several ways.

First, this study is conducted from a new theoretical framework that incorporated information disorder. Little prior research has emphasized the relationship between information disorder and organic food consumption. Although Kushwah et al. (28) constructed an innovation resistance framework and tried to use consumers' organic food involvement to moderate the relationship between innovation barriers (i.e., value, risk and image barriers) and organic food purchase behavior. Nonetheless, the authors only considered the purchase barriers brought by organic food as an innovative product for consumers and did not consider information disorder barriers affecting consumers through technologies such as the internet and social media. The current study found that information disorder is an important peripheral cue for organic consumers.

Second, this paper introduces the concept of information disorder and divides it into three dimensions: organic food information uncertainty, information search anxiety and information overload. The present study was the first attempt to probe into the relationships between information disorder, attitude, involvement and purchase behavior. This study found that the indirect relationship between organic food information uncertainty and purchase behavior is moderated by involvement. That is, involvement weakened the negative effect of organic food information uncertainty on attitude.

Finally, the current study presents the concept of information disorder to the realm of organic consumption, providing valuable insights into which have not been previously studied. The current research is novel in examining the interplay between information disorder and organic food purchase behavior, making an important contribution to organic consumption literature.

### Practical implications

There are three main practical implications of this study. Initially, the rapid social media and internet evolution had effects that have served as a double-edged sword to consumers. Such technologies can facilitate consumers' access to information on organic food. For example, previous studies posited that consumers' access to more information on organic food through the internet or social media can encourage them to buy organic food (15). In addition, the rapid social media and internet evolution has had some negative effects on consumers, particularly in terms of information disorder (16). This study confirmed that information disorder has a significantly negative effect on purchase behavior. Thus, organic food producers, sellers and public departments could formulate corresponding strategies to avoid or reduce information disorder among consumers resulting from internet or social media use. For instance, organic food producers can provide more production information to consumers on authoritative websites or social platforms. Organic food sellers need to establish an organic food advertising system of public trust. At the same time, public departments should strengthen the social media and internet supervision to ensure the authenticity of information provided through these platforms. Second, the indirect relationship between organic food information uncertainty and purchase behavior is moderated by involvement. In other words, involvement weakens the negative impact of organic food information uncertainty on attitude, promoting organic food consumption. As such, organic food producers, sellers and public departments could formulate corresponding strategies to enhance consumer involvement in organic food. For example, organic food producers can establish a good traceability system that can make it more convenient for consumers to access information on organic food production to increase consumers' involvement in organic food. Organic food sellers can promote consumers' involvement in organic food by advertising in important competitions, holding frequent organic food fairs, and carrying out organic food experience activities in combination with rural tourism. Public departments should actively coordinate, organize and guide the establishment of organic food demonstration bases, promote qualified places and enterprises to carry out the establishment of organic food product demonstration areas, strengthen public brand construction of demonstration bases and promote organic food consumption. Finally, attitude is positively associated with purchase behavior. Hence, public departments could establish concepts of organic breeding and planting conducive to ecological civilization by education and publicity and enhance consumers' positive attitude toward organic food to promote its consumption.

## Limitations and further research

The present study has two major limitations. First of all, the study sample was collected from organic consumers in organic food demonstration counties in Jiangxi Province, China, which might limit the generalizability of the research results to other contexts. Expansion of the research to more cities or potential consumers is necessary in the future, which may improve the generalizability of the findings of the study. In addition, this study discussed the impact of information disorder on consumers' organic food purchase behavior. Nevertheless, the study only considered three dimensions of information disorder, that is, organic food information uncertainty, information search anxiety, and information overload. This may not account for the impact of information disorder factors in other dimensions. Future research may take other dimensions of information disorder (e.g., information fatigue) into consideration.

## Data availability statement

The datasets presented in this study can be found in online repositories. The names of the repository/repositories and accession number(s) can be found in the article/Supplementary Material.

## Author contributions

DC shaped the theoretical design. YZ was responsible for the statistical analysis and the final composition. All authors contributed to the article and approved the submitted version.

## Funding

This work was supported by Jiangxi Social Science 14th Five Year Plan fund project (21ST03).

## Conflict of interest

The authors declare that the research was conducted in the absence of any commercial or financial relationships that could be construed as a potential conflict of interest.

## Publisher's note

All claims expressed in this article are solely those of the authors and do not necessarily represent those of their affiliated organizations, or those of the publisher, the editors and the reviewers. Any product that may be evaluated in this article, or claim that may be made by its manufacturer, is not guaranteed or endorsed by the publisher.
