# Supplementary material for: Information disorder and organic food purchasing behavior: A moderated mediation model
Source: Front Nutr. 2022 Jul 19;9:939454. doi: 10.3389/fnut.2022.939454 (PMC9344066; doi:10.3389/fnut.2022.939454)
Supplement: Supplementary file 2 [file DataSheet1.pdf]

Appendix A. Per capita disposable income of demonstration counties in Jiangxi Province in 2019

County	Per capita disposable income （yuan）	Income stratum	Group	County	Per capita disposable income （yuan）	Income stratum	Group
Qingyuan	40160	high	4th	Anfu	31741	moderate	5th
Nanchang	40106	high	4th	Yudu	31373	moderate	5th
Xinjian	39871	high	3rd	Xiushui	30913	moderate	2nd
Guangfeng	39803	high	3rd	Wanzai	30759	moderate	1st
Yuanzhou	38801	high	4th	Lichuan	30458	moderate	3rd
Xiangdong	38672	high	4th	Xingguo	30348	moderate	1st
Guixi	37458	high	2nd	Suichuan	29835	low	4th
Jinxian	37446	high	4th	Xiajiang	29802	low	4th
Jinggangshan	37295	high	2nd	Chongyi	29561	low	3rd
Nancheng	36836	high	4th	Yanshan	28915	low	4th
Dexing	36783	high	1st	Quannan	28751	low	4th
Ruichang	36019	high	4th	Shangyou	28629	low	5th
Luxi	35721	high	5th	Duchang	28586	low	3rd
Nanfeng	35081	high	4th	Guangchang	28566	low	5th
Xingan	35063	high	2nd	Wuyuan	28330	low	2nd
Fengxin	34923	moderate	3rd	Zixi	28053	low	1st
Shanggao	34771	moderate	5th	Tonggu	27949	low	2nd
Yujiang	34754	moderate	3rd	Shicheng	27328	low	2nd
Pengze	34726	moderate	5th	Lianhua	27060	low	2nd
Yifeng	34270	moderate	3rd	Anyuan	26920	low	4th
Yongfeng	33818	moderate	3rd	Yongxin	26798	low	1st
Fuliang	32708	moderate	2nd	Lean	26233	low	3rd
Jingan	32281	moderate	2nd	Poyang	26186	low	5th

Data source: Jiangxi Statistical Yearbook (2020)（www.tjj.jiangxi.gov.cn）
